# All models are wrong, but some organoids may be useful

**DOI:** 10.1186/s13059-019-1677-4

**Published:** 2019-03-27

**Authors:** Benjamin E. Mead, Jeffrey M. Karp

**Affiliations:** 1 Broad Institute of Massachusetts Institute of Technology and Harvard University, Cambridge, MA USA; 2 Institute for Medical Engineering and Science (IMES), Massachusetts Institute of Technology, Cambridge, MA USA; 3 Department of Chemistry, Massachusetts Institute of Technology, Cambridge, MA USA; 4 Koch Institute for Integrative Cancer Research, Massachusetts Institute of Technology, Cambridge, MA USA; 5 Ragon Institute of Massachusetts General Hospital, Massachusetts Institute of Technology and Harvard University, Cambridge, MA USA; 6 Engineering in Medicine, Department of Medicine, Center for Regenerative Therapeutics, Brigham and Women’s Hospital, Harvard Medical School, Boston, MA USA; 7 Harvard−Massachusetts Institute of Technology Division of Health Sciences and Technology, Massachusetts Institute of Technology, Cambridge, MA USA; 8 Harvard Stem Cell Institute, Harvard University, Cambridge, MA USA

Modeling is an essential part of the scientific method. A model enables us to learn about our surroundings by simplifying a complex observation to a set of principles—conceptual or operational—and, in the process, allows for the extension and testing of ‘observable truths’. In the life sciences, biological models have a particularly long history and still play an essential role. They include small animal models that are used widely in genetics and developmental biology, large animal models for therapeutic development, and cellular models that are used to study disease and to develop new medicines.

Cellular models, including cancer-derived cell lines, primary tissue-derived cultures, and induced pluripotent cells and their progeny, are especially ubiquitous thanks to their relative ease of use and scalability. Indeed, cellular models have long served as a backbone of drug discovery, but the lack of novel preclinical models for complex diseases has proven limiting to therapeutic efforts [1]. For every medicine brought to market, countless more agents that were identified in cellular models have failed to provide a therapeutic effect [2]. These ‘misses’ can, in part, be attributed to failures of physiological representation in the existing models. Every model, conceptual or physical, has distinct advantages and disadvantages, and the more widely it’s used, the better it’s understood. To paraphrase a quote that is often attributed to the renowned statistician George EP Box, ‘all models are wrong, but some are useful’. It’s not a leap to question whether we might have fully extracted the therapeutic discovery potential of the cellular model.

Fret not—thanks to efforts in stem cell biology, bioengineering, and the like, new cellular models with promise of improved representation are arising at an impressive rate. But this does not free us from Dr. Box’s truism, and we must still consider why a particular model is best suited to our most pressing questions. This reasoning may seem intuitive, but it is often countered by the scientific ‘hype cycle’, in which new models, approaches, and ideas are over-promoted for their novelty, often before they are fully appreciated.

Organoids, a label adopted to define a plethora of cellular models that have arisen in the past decade, may now be in a moment of ‘hype’ [3]. We loosely define organoids as three-dimensional, self-organizing, stem-cell-derived structures that resemble their in vivo tissue counterparts [4]. For example, an intestinal organoid is conventionally formed from isolated adult LGR5+ intestinal stem cells, which are seeded in an ill-defined complex extracellular matrix enriched with a growth factor-laden medium to recapitulate the in vivo stem-cell niche. In just a few days, an ordered multicellular assembly resembling, to a degree, the in vivo intestinal epithelium appears. This intestinal organoid can be maintained in culture, with passaging, for a potentially indefinite period. Organoids promise greater representation of our tissues when compared to cell lines, but offer reduced complexity when compared to tissue explants or animal models. As more human- and animal-derived organoid models become available (representing the gastro-intestinal tract, liver, pancreas, kidney, heart, brain, and tumors), researchers have increasingly turned their focus to applying organoid models to the study of a broad swath of human disease, often leveraging new and improving experimental approaches (including gene-editing, high-throughput screening, and multi-omic profiling) to assess the cellular state comprehensively [5].

Organoid models have provided useful insights, beyond the capabilities of the preceding cellular models, into the heterogeneities of cancer, diseases of hereditary genetics (such as cystic fibrosis), and host–pathogen interactions (with regard to norovirus and Zika virus) [6]. A closer look at many of these studies reveals a commonality between the modeled phenotype, and intrinsic elements that are often measured in many cellular models, including organoid systems: proliferation and gene-driven phenotypes. What is new is the ability to capture human patient-specific manifestations and to recapitulate the dynamics of tissue development. Nevertheless, the application of organoid models to complex diseases that are not necessarily linked to tissue development or monogenetic drivers has been limited. In such instances, the subtler phenotypes, such as those present in many polygenic autoimmune conditions, may not manifest if the originating cell state or pertinent environmental cues in vivo are not accurately represented within an organoid model. Put simply, we have not mapped the broad space of the advantages and disadvantages of an organoid model applied to human disease, and should be especially diligent in our application of organoid models.

Our lab [7] and others [8] have begun to conduct experiments to explore approaches for assessing and improving cellular fidelity in intestinal organoids. We have observed that a ‘conventional’ small intestinal organoid provides only a limited representation of the Paneth cell, a mature cell type of the small intestine. By rationally changing the culture environment, we can produce a higher-fidelity model of that cell type, but in doing so we sacrifice ‘total’ representation of the multiple cell types of the epithelium. This would suggest that although a conventional intestinal organoid has signatures that represent all cell types of the epithelium, it does not have complete, functional representation of each cell type. Therefore, more suitable models may exist for investigations of a specific cell type. This exploration is really just the tip of the iceberg in defining the space beyond the developmental uses of organoid models, and shows that we must suitably validate our models to specific questions, by first understanding the minimal set of cell types and the interactions that must be present to replicate a phenotype.

Questions that aim to dissect complex phenomena, which are involved in almost any study of human disease, require nuanced models, including organoids. To apply an organoid model properly, however, we must conduct a critical assessment of whether the problem (or hypothesis) is well-defined, and we must incorporate existing knowledge of the phenotypic aspects that are recapitulated in organoids. Further, it is important to rationalize why an organoid model is preferred when a cell line may give the same result. Many potential questions remain to inform this type of assessment, including:

- To what resolution might we be able to model manifestations of disease in an organoid? Does the model provide only measures of cellular survival, or will its fidelity extend to molecular measures that can be read with multi-omic tools?
- If we can derive organoid models from individuals with complex disease, what are the ‘bounds’ of fidelity at the genetic, epigenetic, environmental, and niche levels? Which organoids will retain a memory of disease in vitro [9], and will an organoid be able to manifest the major functional characteristics of disease?
- In diseases with varying degrees of multi-tissue (e.g., involving immune cells), multi-system (e.g., involving paracrine signals), and multi-organism (e.g., gut microbiota) involvement, what aspects of disease can we realistically seek to model in organoids, and how do we think about systems-level interactions or the significant elements that may be missing?
- How might we approach the ‘tuning’ of an organoid model [10] to address an appropriate hypothesis in the study of disease, and where may we apply organoids most effectively in the process of therapeutic development? Are organoids best used as a better system for screening, as a more representative system in which to learn about molecular mechanisms, or to understand off-target effects?

We should embrace systematic approaches to establish the advantages and disadvantages of organoid models. In addition, we should provide an environment for those rigorous experiments to be published, even in the cases where they may contradict the use of a model. As with the establishment of any new model, we should balance the excitement of future utility with a frank discussion of the hurdles that must be addressed to realize that promised utility. This is the time to question the most, because often the science can advance quickly and people will accept statements before they gain a firm understanding of the evidence. To realize the full potential of an organoid model in the study of human disease and the ultimate benefit to society, we are all obligated to make sure the foundation is solid—and this requires careful and rigorous interrogation.
